# Outdoor Activity Participation Improves Adolescents’ Mental Health and Well-Being during the COVID-19 Pandemic

**DOI:** 10.3390/ijerph18052506

**Published:** 2021-03-03

**Authors:** S. Brent Jackson, Kathryn T. Stevenson, Lincoln R. Larson, M. Nils Peterson, Erin Seekamp

**Affiliations:** 1 Fisheries, Wildlife and Conservation Biology Program, College of Natural Resources, North Carolina State University, Raleigh, NC 27695, USA; mnpeters@ncsu.edu; 2 Department of Parks, Recreation and Tourism Management, College of Natural Resources, North Carolina State University, Raleigh, NC 27695, USA; kathryn_stevenson@ncsu.edu (K.T.S.); lrlarson@ncsu.edu (L.R.L.); elseekam@ncsu.edu (E.S.)

**Keywords:** COVID-19, adolescents, subjective well-being, resilience, mental health, outdoor activities

## Abstract

COVID-19 is reshaping human interactions with the natural environment, potentially generating profound consequences for health and well-being. To assess the effects of COVID-19 on the outdoor recreation participation and subjective well-being of adolescents, as well as how participation in outdoor activities may mitigate declines in subjective well-being, we used a Qualtrics XM panel to conduct a nationally representative survey of youth ages 10–18 across the United States (*n* = 624) between 30 April and 15 June 2020. Survey questions focused on frequency of participation in outdoor activities before and during the pandemic, as well as changes in subjective well-being. Paired *t*-tests revealed decreases in both outdoor recreation participation (64% reported declines) and subjective well-being (52% reported declines). A regression model examining correlates of changes in subjective well-being (*R*^2^ = 0.42) revealed strong associations with changes in outdoor play (*B* = 0.44, *p* < 0.001) and nature-based (*B* = 0.21, *p* = 0.016) activities. Adolescents’ from all backgrounds who participated in these activities during the pandemic reported smaller declines in subjective well-being. Results highlight the critical role that time outdoors and time in nature play in bolstering adolescents’ resilience to stressors such as the COVID-19 pandemic and underscore the need to facilitate outdoor recreation opportunities for youth during times of crisis.

## 1. Introduction

Global change threatens the resilience of socio-ecological systems, including human health and well-being. Impacts from land-use change, climate change, ecosystem degradation, and global health crises are evident in the increased risk of exposure to infectious disease, water scarcity, food scarcity, natural disasters, and population displacement [1]. In the context of human health, resilience can be defined as the ability to maintain a high-level of well-being by coping and adapting to adverse social and environmental changes [2,3,4,5]. Subjective well-being (SWB), defined as a sense of life satisfaction, positive affect, and low negative affect [6,7], is one measure of mental health that may promote resilience to these challenges [2,3,8,9]. Often referred to as a measure of happiness [10], SWB is recognized as the primary measure of hedonic well-being [7] and is frequently employed as an indicator of mental health [11,12]. Understanding the factors that contribute to SWB during a crisis is an important step in identifying strategies to build resilience as global change progresses. Adolescents (youth who are 10–19 year olds [13]) may be particularly susceptible to the impacts of global change as they are acutely impacted by stressors associated with emergencies and disasters [14]. Accordingly, understanding factors that contribute to maintaining the SWB of adolescents in times of stress may be important for promoting the resilience of current and future generations in the face of global change.

The COVID-19 pandemic has become a profound human health stressor associated with global change. While the direct effects of contracting COVID-19 can result in a range of physical health complications including death, the virus also impacts the mental well-being of those not infected [15,16,17,18]. Health initiatives such as physical or social distancing and quarantine intended to curtail the spread of COVID-19 require people to refrain from activities deemed non-essential. While these practices are necessary for protecting public health [19], they put additional stress on adolescents by changing routines and reducing social interactions during a key life stage [20]. Research on the mental health impacts of the 2009 H1N1 pandemic found that nearly one third of youth who experienced isolation or quarantine met the criteria for a PTSD (post-traumatic stress disorder) diagnosis [21]. Preliminary reports on the mental health impacts of COVID-19 in China highlight a rise in psychological disorders, increased anxiety, depression, and stress [22,23,24]. Following a pandemic, a diagnosis of a psychological disorder in a parent is frequently mirrored in their children [21]. These are concerning developments, as the impact of stress on adolescents has been identified as having lasting repercussions, including greater susceptibility to stress later in life [25].

Participation in outdoor activities has potential for bolstering adolescent’s resilience to environmental stressors, including those associated with COVID-19 [26]. Differences between outdoor activities likely have an impact on their effects on SWB. Exposure to nature is one key aspect of participation in outdoor activities that provides a range of health benefits, including relief from stress [27,28,29,30,31,32]. Nature may promote resilience among adults by facilitating restoration from stress [33] and buffering against negative health outcomes associated with stress [34]. Health benefits associated with exposure to nature include improved physical health [35,36], but the majority of the findings center on improved mental well-being [37,38,39,40,41,42,43]. Although most of the aforementioned studies focus on adult subjects, exposure to nature may have an even more profound effect on children and adolescents [44,45].

Outdoor activities also provide adolescents with an opportunity to engage in physical fitness, which plays an important role in maintaining physical and mental well-being [46,47]. Research exploring life satisfaction points to declines in participation in physical activity as being linked to declines in life satisfaction [48,49,50]. Previous research also demonstrates that an increase in the frequency and duration of moderate-intensity physical activity is positively associated with SWB [51]. Emergent research aimed at exploring physical activity during COVID-19 suggests children and adolescents may be spending less time engaging in physical activities and more time engaging in sedentary activities [52], which have been shown to negatively affect the physical and mental well-being of adolescents [46]. Studies exploring connections between physical activity and exposure to nature demonstrate that these two factors work synergistically to provide greater positive impacts on physical and mental health than physical activity alone [53], highlighting the potential benefits of adolescent outdoor activities that incorporate physical activity in the form of outdoor play.

Outdoor activities also play a pivotal role in the development and maintenance of social capital and cohesion, which can influence mental health for both adolescents and adults [54]. Individuals who are socially isolated are physically and psychologically less healthy [54] and more susceptible to stress [55]. Research on the role of social capital in promoting the use of green space and physical activity points to social relationships as a key factor encouraging the use of green space [56] and participation in physical activities [57]. Social interactions with immediate family have been identified as particularly beneficial, with increases in time spent with family resulting in increased SWB [58]. Within the context of COVID-19, adolescents are primarily limited to social interactions with direct family members, demonstrating the potential importance of family-centered outdoor activities that provide social interaction, build social capital, and facilitate exposure to nature and physical fitness.

Given these benefits of outdoor recreation, early reports suggesting the COVID-19 pandemic has reduced participation in outdoor activities [18,52] are troubling. Yet, the negative trends in participation rates can be responsibly reversed since exposure to the virus is less likely in outdoor spaces compared to indoor spaces [59]. Prior to the pandemic, adolescents could participate in a variety of recreational activities through community recreation programs, schools, and organized sports [60]. Participation in these activities expose adolescents to nature, physical activity, and social interactions that can generate multiple health benefits [28]. During the pandemic, however, these activities have likely been impacted by social distancing guidelines, potentially limiting adolescent’s ability to benefit from health-buffering factors during a time of increased stress [16]. In many cases, outdoor activity participation may have decreased due to school, park, and outdoor recreation space closures, as well as cancelled team sports and activity classes [52,61]. However, evidence suggests that other outdoor activities (e.g., neighborhood walks, visiting local parks) may have increased as some people seek ways to get out of their homes and safely interact with others [62].

While the stress of COVID-19 is likely negatively affecting all adolescents to some degree [63], some are at a greater risk from the effects of the pandemic than others [64]. For instance, health data reveal higher COVID-19 infection rates in Black communities compared to other demographic groups [65,66]. Disparities in infection rates may be exacerbated by disparities in outcomes from social distancing efforts such as intensifying food insecurity and declining educational outcomes among underserved communities when schools are moved online [64]. Prior to the pandemic, girls, Black youth, and older adolescents spent less time outside and more time on electronic devices, highlighting potential trends that may persist during the pandemic [67]. Additionally, adolescents in urban environments are at a higher risk for viral infection, and there are fewer opportunities for exposure to nearby nature [26,68], which has been identified as a significant factor in children’s ability to cope with stress [44,45]. The perceived quality of nearby nature has also been shown to play an important role in the effectiveness that time outside has on mental well-being, highlighting additional inequities in opportunities for exposure to the benefits of time in nature [69]. Moreover, the greater density of people seeking nearby nature in urban areas may further restrict access to outdoor recreation opportunities due to social distancing policies, park closures, and citywide lockdowns in areas with high infection rates [61,70].

Characterizing changes in outdoor activity participation, across landscapes and demographic groups (age, race, household-income, community type, and region of the country), is critical to understanding the potentially inequitable impacts of COVID-19 on adolescents SWB. We explored answers to these questions with a nationally representative survey that measured adolescent outdoor activity participation and SWB both before and during the COVID-19 pandemic. We tested several hypotheses. First, (H1) we predicted that adolescent SWB decreased as COVID-19 emerged, likely due to the wide array of stressors associated with a global health crisis [14,21,71,72]. Next, (H2) we hypothesized that adolescent participation in outdoor activities (outdoor play activities, nature-based activities, and outdoor family activities) decreased during the COVID-19 pandemic [52]. Finally, we expected to see relationships linking participation in outdoor activities with higher levels of SWB. As time outdoors may buffer against stress, we hypothesized that (H3) adolescents with high outdoor activity participation levels pre-COVID-19 experienced a smaller decrease in SWB, and that (H4) adolescents who maintained higher levels of participation in outdoor activities during COVID-19 experienced a smaller decline in SWB.

## 2. Materials and Methods

### 2.1. Data Collection

The sample for this study was prepared using an online panel provided by Qualtrics XM through a stratified convenience sampling approach. We chose to use a Qualtrics panel because it allowed for demographic quotas and, when compared to other online panel providers, Qualtrics samples come closest to a national probability sample in terms of demographic representativeness [73]. Qualtrics also allows for rapid data collection—a critical need in our COVID-19-focused study—as it compiles panel respondents recruited from a range of other firms [73]. The Qualtrics panel provided for this study drew from a national pool (50 states, Puerto Rico) with demographic quotas for gender (male, female, non-binary and other), race (White, Black, Hispanic, Asian/Pacific Islander, Native American, other), and community type (rural area, small city or town, suburb near a large city, and large city) representative of the 2019 U.S. census data. Sampling was restricted to parents and their children between the ages of 10–18 years old. We chose this age range because adolescents are particularly susceptible to stress linked to global health crises [14,74], and old enough to understand the survey.

Data collection began 30 April 2020 and closed 15 June 2020. Data were collected through separate but linked parent and child survey instruments that were created and administered using the Qualtrics platform. Surveys were administered to qualifying parents who completed the parent version of the survey before being prompted to hand their device to their qualifying child to complete the adolescent version of the survey. Prior to starting the survey, parents were provided with a linked and downloadable consent form acknowledging their consent to participate and their consent for their child to participate. Adolescents were also provided with an age appropriate assent form acknowledging their consent to participate.

### 2.2. Survey Instrument

The adolescent survey instrument included 21 self-reported items comprising four main constructs, pre and post COVID-19 SWB, pre and post COVID-19 mental health, pre and post COVID-19 outdoor activity participation, and a single item eliciting information about the causal relationship between outdoor activity participation and SWB. Within the context of this study pre COVID-19 refers to the period before the virus impacted the daily lives of respondents, whereas post COVID-19 refers to the period when the survey was completed (1–3 months into the pandemic). In addition to these constructs, adolescents were also asked demographic questions including age, gender, and race. Demographic information gathered from the parent survey included household income, community type, and state of residence.

The four-item SWB construct used for this study was a modified version of the World Health Organization’s (WHO) five-item subjective health and well-being scale [75,76,77], which has been used internationally for measuring the SWB of both children and adults [77]. The scale represents a unidimensional measurement of health with high predictive validity [77]. We made several careful modifications. First, as we were interested in SWB before and during COVID-19, we modified the question stem to assess respondents’ health prior to being asked to practice social distancing as well as after: “How did you feel both before and after you were asked to practice social distancing because of the coronavirus outbreak?” In addition, as this survey was aimed at adolescents, we omitted one item and modified the wording on the remaining items to be appropriate for younger audiences (see Table 1 for final item wording). Lastly, to reduce the burden on respondents, we modified the response items to be four point Likert scales including the responses “at no time”, “some of the time”, “most of the time”, and “all of the time”. While measures of SWB might be impacted by the momentary mood of the respondent at the time of their response, previous research highlights that the use of a multi-item scale is less susceptible to such distortion [78,79]. Measures of recalled mood and emotions are relatively stable and reliable over periods of time ranging from 2 weeks to 2 months [78,80], which was just short of the approximate time frame required for adolescents to recall pre-pandemic SWB in our study. Although acute events experienced by individuals (e.g., getting a bad grade/marks on a test) may impact reported SWB, these individual events do not impact inferences drawn from the overall sample unless they are experienced systematically by relatively large numbers of respondents.

The pre and post COVID-19 self-rated mental health construct we used was a modified version of the scale used in the Behavioral Risk Factor Surveillance System survey [81]. The scale represents an efficient indicator of mental health and has been used to assess population mental health as well as the risk of adverse mental health outcomes [82]. Our version of the scale was modified to assess respondents’ health prior to being asked to practice social distancing as well as after: “How would you rate your health both before and after you were asked to practice social distancing because of the coronavirus outbreak?” The response items for this scale comprised five point Likert scales including “Terrible, Poor, Average, Good, and Excellent”.

Outdoor activity items were focused on determining frequency of participation in specific outdoor and nature-related activities. Adolescents were asked “How often did you participate in the following activities this time last year and now, after you have been asked to practice social distancing because of the coronavirus outbreak?” Both the retrospective and current iteration of the items used a three point Likert scale with the responses “Never”, “Every now and then”, and “Often”. A short response scale was used for this construct as our research questions are focused on determining directional trends rather than specific measures of intensity or extremity [83]. We included five “outdoor play” activities that could be done in any type of outdoor environment (playing sports outside, bicycling outside, going for walks or runs outside, swimming outside, skating), eight “nature-based activities” confined to more natural settings (camping, wildlife viewing, hiking, paddling, hunting, fishing, playing in the woods, collecting natural items), and a single item measuring “outdoor family activities” (spending time with my family outdoors), for a total of 14 different activities. These activities were selected based on retrospective qualitative interviews conducted with young adults (18–35 years old) during the summer of 2019. During these interviews respondents shared the childhood experiences that shaped their connection to nature. Activities were also selected based on previous studies focused on adolescent participation in outdoor and physical fitness activities [49], including those that noted the importance of distinguishing between outdoor play and nature-based activities [84]. We also included a more general outdoor activity item that used the same question stem as the previous outdoor activity items but referred to participation in “some sort of outdoor activity” rather than a specific activity. Both the retrospective and current aspects of this item used a five point Likert scale consisting of the responses “less than one time per month”, “1–2 times per month”, “1 time per week”, “2–4 times per week”, and “5 or more times per week”.

The single item: “Has spending time outdoors in nature helped you deal with the stress caused by practicing social distancing because of the coronavirus outbreak?” was also included in order to assess face validity of a causal relationship between outdoor activity participation and SWB. The item included a five point Likert response scale comprised of “Not at all”, “Somewhat”, “Definitely”, and “Does not apply, as I haven’t spent much time outdoors since I was asked to practice social distancing”.

### 2.3. Data Analysis

#### 2.3.1. Data Preparation

We used listwise deletion to remove 257 responses that were either straight-line responses (answering the same for all questions) or nonsensical text responses (related to open text questions), resulting in a final sample of 624. When a survey response was removed from the sample, the corresponding parent or child survey was also removed. Parent and child surveys were linked using Qualtrics embedded dyad codes. All items were analyzed based on coding described above with the following exceptions. The response scale for the general outdoor activity item was recoded so that “less than one time per month” = 0.25, “1–2 times per month” = 0.5, “1 time per week” = 1, “2–4 times per week” = 3, and “5 or more times per week” = 5. We recoded these values to approximate the actual number of outdoor activities adolescents participated in during the week. The response scale for the item “Has spending time outdoors in nature helped you deal with the stress caused by practicing social distancing because of the coronavirus outbreak?”, was also recoded so that the responses “Not at all” and “Does not apply” were grouped together as “No”, while the responses “Somewhat” and “Definitely” were grouped together as “yes”. This helped to streamline the analysis and clarify directionality of the relationship between outdoor activity participation and SWB. Children identifying as more than one race were grouped into a single “two or more races” category. State of residence data were broken into 4 geographic regions delineated by the U.S. Census Bureau, with Alaska and Hawaii being added to the West region and Puerto Rico being added to the South region (South: AL, AR, DC, FL, GA, KY, LA, MD, MS, NC, OK, PR, SC, TN, TX, VA, WV) (Northeast: CT, DE, ME, MA, NH, NJ, NY, PA, RI, VT) (Midwest: IL, IN, IA, KS, MI, MN, MO, NE, ND, OH, SD, WI) (West: AK, AZ, CA, CO, HI, ID, MT, NV, NM, OR, UT, WA, WY) [85]. The cleaned dataset was analyzed with Stata 14.1.

#### 2.3.2. Activity Grouping and SWB Scale Analysis

We used exploratory factor analysis (principal component factor analysis, or PCF) with an orthogonal varimax rotation to assess the dimensionality and internal consistency of our modified four-item WHO SWB scale (Table 1). The analysis supported a unidimensional factor structure that explained 70% of the variance. The scale also demonstrated high internal consistency (α = 0.852) and acceptable convergence (all items loaded with eigenvalues >0.8). We selected outdoor recreation activities for each grouping a priori, and assessed the validity of these groupings using PCF to examine the structure of all individual pre COVID-19 activities (Table 2). The analysis supported a two-factor structure explaining 55% of the variance. These factors were outdoor play activities (5 items, α = 0.784), and nature-based activities (8 items, α = 0.876). The single-item outdoor family activities was also included as an activity group although it was not included in the factor analysis. Both activity groupings displayed acceptable convergence (all activities loaded with eigenvalues > 0.5). We created composite scores for each activity grouping by averaging responses.

#### 2.3.3. Hypothesis Testing

To address our first two hypotheses, we used paired sample t-tests to compare pre- and post-COVID-19 levels of SWB and self-reported mental health. We also used paired sample *t*-tests to compare pre and post COVID-19 activity scores for the three types of outdoor activities (outdoor play, nature-based, and outdoor family) and general outdoor activity participation. We used the Bonferroni correction to address family-wise error rates associated with conducting multiple tests of significance [86]. To evaluate our third and fourth hypotheses exploring the relationship between outdoor activity participation and change in SWB, we used a multiple linear regression model. We modeled change in SWB for each adolescent respondent as a function of their pre outdoor play activity score, pre nature-based activity score, pre outdoor family activity score, change in outdoor play activity score, change in nature-based activity score, and change in outdoor family activity score. Our model also included the pre-COVID-19 SWB score to control for a potential ceiling effect where respondents with low initial SWB scores have less room for declines than those with high initial SWB scores [87]. We also controlled for household income, gender (with males as the reference group), race (with White as the reference group), community type (with suburbs near a large city as the reference group), and geographic region (with South as the reference group). We selected these reference groups as they represent the groups with the highest sample size in their respective categories. We conducted a post hoc power analysis of our multiple linear regression using the G*power 3.1 statistical package [88]. This test yielded a value of approximately 1.00 for the power of the omnibus *F* test, indicating a near 0% chance of a false negative result.

To explore the potential for a causal relationship, we used a one-way ANOVA to model change in SWB by perception of whether outdoor activity participation helps with stress. We also ran a second one-way ANOVA modeling change in self-reported mental health by the same outdoor time and stress question.

## 3. Results

### 3.1. Sample

Our sample (*n* = 624) was comprised of an equal gender ratio, was 59.8% White, and included adolescents ranging from 10–18 years old with relatively equal splits across ages. Household income was normally distributed and the Southern region of the United States had the greatest number of respondents, with suburbs of large cities being the most common community type (Table 3).

### 3.2. Subjective Well-Being and Mental Health Scores

We found support for H1 as adolescents reported a 23.0% decline in SWB scores (pre-COVID-19 *M* = 2.21, *SD* = 0.62; post-COVID-19 *M* = 1.75, *SD* = 0.75; *t*(623) = 14.87, *p* < 0.001; Table 4) and a 9.3% decline in self-reported mental health scores during the pandemic (pre COVID-19 *M* = 4.31, *SD* = 0.80; post COVID-19 *M* = 3.92, *SD* = 0.96; *t*(623) = 10.92, *p* < 0.001; Table 4). Overall declines in SWB were reported by 51.6% of adolescents, with 6.1% reporting increases. Overall declines in self-reported mental health were reported by 34.9% of adolescents, with 6.7% reporting increases.

### 3.3. Outdoor Activity Scores

**Figure 1:**
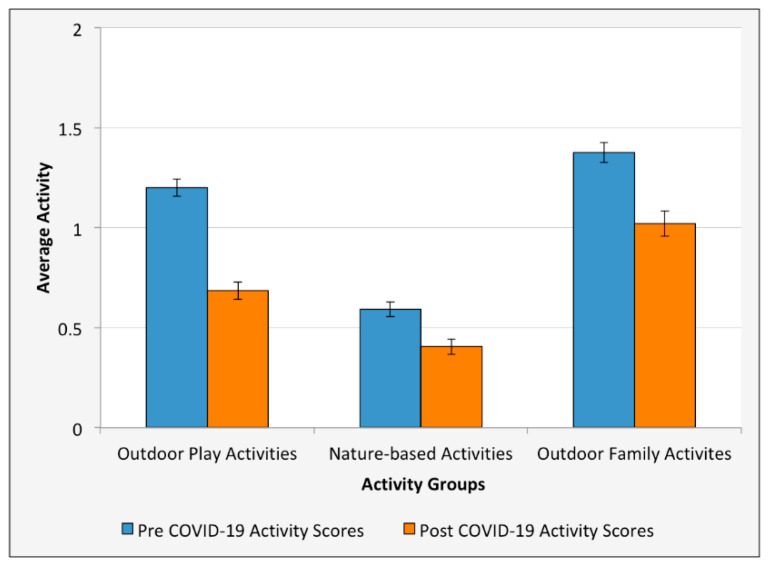
Changes in outdoor activity participation rates (by type of outdoor activity) pre- and post-COVID-19 pandemic for adolescents in the United States (*n* = 624). Mean activity scores ranged from 0 (never participate) to 2 (often participate).

Declines across all outdoor activity groups support H2 as outdoor play activities dropped by 41.6%, nature-based activities dropped by 39.7%, and outdoor family activities dropped by 28.6% (Table 4, Figure 1). Declines in general outdoor activity participation during the pandemic were reported by 52.4% of adolescents, resulting in a 21.6% decrease in outdoor activity participation (pre COVID-19 *M* = 3.68, *SD* = 1.17; post COVID-19 *M* = 2.89, *SD* = 1.45; *t*(623) = 11.82, *p* < 0.001; Table 4). During COVID-19, 59.9% of adolescents reported participating in an outdoor activity once per week or less, 40.2% participated once every two weeks or less, and 27.4% participated once a month or less.

### 3.4. Linear Regression Model

We found partial support for H3, as individuals who participated in more outdoor play activities pre COVID-19 were more resistant to negative changes in their SWB score during the pandemic (*B* = 0.30, *p* < 0.001; Table 5). We did not detect relationships between pre-COVID-19 activity levels and change in SWB associated with nature-based or outdoor family activities.

**Figure 2:**
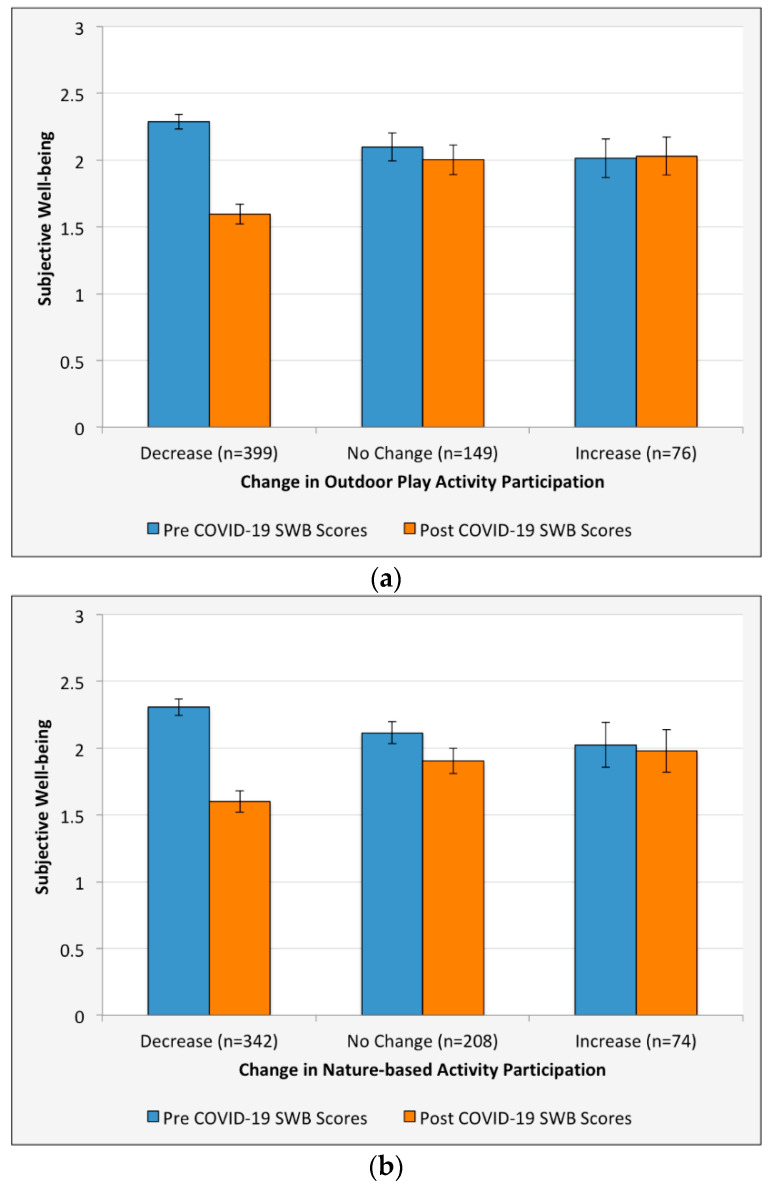

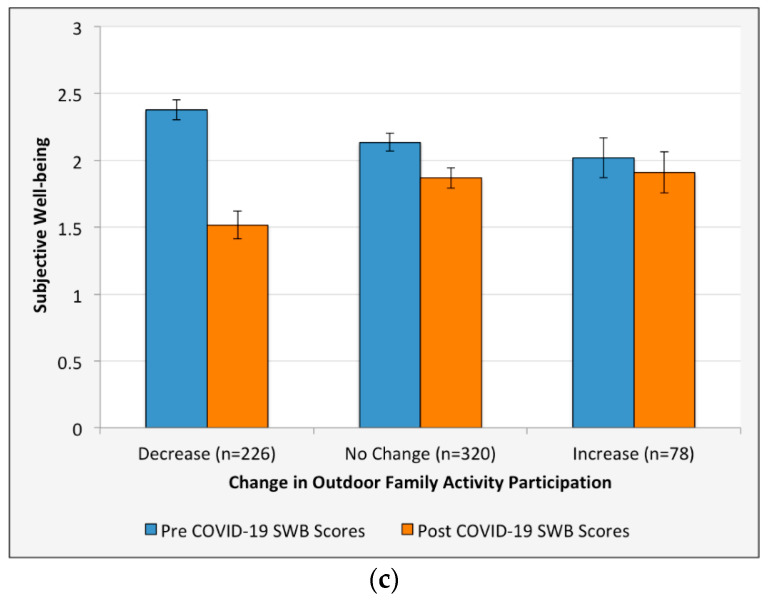
Changes in subjective well-being scores by changes in rates of outdoor play activity (**a**), nature-based activity (**b**), and outdoor family activity (**c**) participation pre and post COVID-19 pandemic for adolescents in the United States (*n* = 624). Mean activity scores ranged from 0 (at no time) to 3 (all of the time).

We found partial support for H4, as declines in outdoor play and nature-based activities during the pandemic were associated with declines in SWB scores. Continued participation in outdoor play activities (*B* = 0.44, *p* < 0.001; Table 5, Figure 2a) and nature-based activities (*B* = 0.21, *p* = 0.016; Table 5, Figure 2b) buffered adolescents against the negative impacts of COVID-19 on SWB. For both of these activity groups, high levels of participation were associated with post-COVID-19 SWB levels approximating those experienced in a pre-COVID-19 context. We did not detect a relationship between participation in outdoor family activities and SWB (*B* = 0.06, *p* = 0.123; Table 5, Figure 2c), but the non-significant relationship had the same valence as those detected for other activity types. The relationship between outdoor activity participation and SWB was positive for all activity groups both before and after COVID-19. Demographic variables (gender, age, race, household income, community type, and geographic region) were not significant in this model, however effects appeared to be slightly more magnified for adolescents in urban communities (*B* = −0.10, *p* = 0.140).

During the pandemic, 76.4% of adolescents reported that spending time outside in nature helped them deal with the stress caused by practicing social distancing. Furthermore, the adolescents who said time outdoors helped them cope with pandemic-related stress reported less pronounced declines in SWB (*M* = −0.39, *SD* = 0.73) than those who did not recognize these benefits (*M* = −0.70, *SD* = 0.87) (*F*(1622) = 17.72, *p* < 0.001). Similar patterns were observed with respect to self-reported changes in mental health: adolescents who said time outdoors helped them cope reported less pronounced declines in SRMH (*M* = −0.35, *SD* = 0.89), than those who did not recognize these benefits (*M* = −0.54, *SD* = 0.90) (*F*(1622) = 5.18, *p* = 0.023).

## 4. Discussion

Our study revealed declines in SWB and outdoor activity participation that may be casualties of community health initiatives aimed at reducing the spread of COVID-19. Adolescents’ SWB dropped during the pandemic, as did participation in outdoor activities. Adolescents with high participation rates in outdoor play activities prior to the pandemic had smaller decreases in their SWB, and those that continued to participate in outdoor play and nature-based activities during the pandemic were buffered against declines in SWB. Adolescents who reported that spending time outdoors in nature helped them deal with the stress associated with the pandemic experienced smaller declines in both their SWB and their self-reported mental health.

Declines in adolescents SWB identified in this study highlight an underlying and largely unexplored COVID-19 related health risk. Our SWB findings support previous research pointing to the negative impacts of pandemics, natural disasters, and large-scale emergencies on the mental health of adolescents [14,21], further elucidating risks posed by the expanding scale and frequency of global change events [1]. This is concerning, as decreases in adolescents SWB hamper social, emotional, and academic development [89]. Additionally, an increase in stress and trauma at a young age can have long-term impacts that affect SWB later in life [25,90], and may lead to other health disorders [91], hinting at the potential for a health crisis that may unfold for years to come. The potential impacts of declines in SWB measured in this study lend further support to the importance of identifying and promoting resilience-enhancing factors that allow adolescents to better cope and adapt to global change events [4].

The decline in adolescents’ outdoor activity participation may be an artifact of where and how adolescents engage in outdoor activities, as pandemic related closures reduce access to recreation spaces and remove outdoor activities built into daily routines. Some outdoor play and outdoor family activities were likely accessible to adolescents before and during the pandemic, as they can be conducted near home while maintaining social distancing. Despite this, concerns regarding the safety of all outdoor activities may have contributed to the decline in participation, as well as closures or overcrowding in available public outdoor spaces [18,61,70]. Safety concerns coupled with the loss of structured recreation opportunities (e.g., school sports) may help explain the large decline in outdoor play activities. Other research has indicated a drop in physical activity and a rise in sedentary behaviors when school is not in session [92]. The smaller decline in outdoor family activities may be attributed to the broad nature of this outdoor activity group, as well as the relative safety of recreating with family units versus the risks associated with interacting with other individuals. Declines in nature-based activities could be explained by limited access to natural areas due to park closures [70] and the increased risk associated with traveling further to reach natural areas [93]. The low participation rates in nature-based activities versus other activity groups even before the pandemic, may also point to barriers such as access to natural areas, which may have been exacerbated during the pandemic [61]. Declines across all outdoor activities identified in this study represent disturbing trends with potentially long-term adverse effects [52], as adolescence is a key life stage where lifestyle habits develop and shape outdoor recreation patterns and preferences in adulthood [94,95,96]. Offering and promoting recreational opportunities that facilitate COVID-19 appropriate outdoor activities at or near home (e.g., keeping municipal park spaces open, closing city streets for pedestrian use) may improve participation rates [70,97], particularly while recreation opportunities adolescents routinely participate in (e.g., school programs, organized sports, clubs and summer camps) are unavailable. Such programs could build on other research highlighting the health benefits of “nearby nature” and outdoor recreation experiences [44,45,98].

Our results indicate that frequent participation in outdoor play activities prior to the pandemic provided lasting resilience against drops in SWB during the pandemic. Several studies with adults suggest that regular outdoor recreation may provide mental resiliency to stress [18,99]. For instance, an experimental study in the United Kingdom found that adults participating in a 10-week outdoor walking program had improved mental health for at least one year [100]. Another study found that regular outdoor recreation in both neighborhoods and nearby natural areas was associated with long-term well-being and psychological resilience [99]. Our results indicate that similar trends may hold for adolescents, with those who participate in frequent outdoor play having increased resiliency to declines in SWB under stress. Future research should continue to explore this possibility, as well as measure, and mitigate detrimental impacts of pre-existing outdoor play deficits on SWB [101]. As continued participation in each of the outdoor activity groups provided some relief from negative impacts on SWB during the pandemic, adolescents may reap the benefits of outdoor activity participation regardless of pre COVID-19 outdoor activity participation.

Differences in levels of exposure to nature, physical activity, and social interactions between outdoor activity types may account for the variation in each activity’s capacity to buffer against declines in SWB during the pandemic. Continued participation in outdoor play and nature-based activities during the pandemic buffered adolescents against declines in their SWB, resulting in post COVID-19 SWB scores similar to pre COVID-19 scores. Outdoor play was particularly effective at reducing the decline in SWB, nearly doubling the efficacy of nature-based activities. Outdoor play activities tend to be more accessible than nature-based activities, and are therefore engaged in more frequently. These activities also provide the potential for exposure to nature and are often more physical fitness-oriented. Prior to COVID-19, outdoor play activities also provided adolescents with opportunities for social interaction [56,102]. Social interactions likely persist during the pandemic, but they may be limited to family and small groups of neighbors [62]. In contrast, participating in nature-based activities during the pandemic is less frequent for adolescents and may be limited to immediate family. Outdoor family activities are not mutually exclusive and can include any of the other activities but within a family context. However, the weaker relationship between outdoor family activities and SWB may be due to being isolated with family during the pandemic, resulting in an increased amount of time being spent together, which some studies have shown has heightened family-level stress in the context of the COVID-19 pandemic [103,104]. Accordingly, although outdoor family activities are important [54], within the context of COVID-19 it may be beneficial to spend time away from family. These differences between activity groups help to explain the effectiveness of outdoor play activities during COVID-19 and demonstrate the potential of nature-based and outdoor family activities for improving SWB outside of the COVID-19 context.

Our findings suggest COVID-19 negatively impacts adolescents SWB and outdoor activity participation regardless of race, gender, age, household income, community type, and geographic region. Further, we did not find significant disparities in activity participation or SWB based on these demographics. We find this latter result particularly surprising and encouraging, given the well-documented disparities in both access to nature [68,105] and more serious health impacts of COVID-19 felt by Black, Hispanic, and other racial and ethnic minority communities [65]. However, restrictions on outdoor activities related to COVID-19 have been largely geographic rather than demographic (e.g., entire states imposing mobility restrictions), and previous research indicates that demographic variables have a relatively weak impact on life satisfaction compared to environmental factors [106]. Although all adolescents have been affected by COVID-19, outdoor activity-focused interventions might help promote SWB for all, demonstrating a need to promote adolescent participation in such activities and increase equitable access to nature and recreational spaces [107,108]. This appears to be particularly true for nature-based activities, as pre COVID-19 participation rates were less than half that of the next closest activity group. Initiatives such as Blue Sky Funders Forum’s Rethink Outside [109] and Sierra Club’s Outdoors for All [110] are working towards this goal, but additional research highlighting nature as essential to human health and well-being is needed to leverage the potential of such initiatives.

### Recommendations for Future Research

This study highlights a need for additional research exploring the potential for outdoor activities as a means of building adolescents resilience against global change events. Future studies should continue to aim for large, representative samples and consider including respondents outside of the United States. These additions would highlight pandemic impacts on youth in other regions and illustrate how trends found here may hold or change across cultural contexts. Additionally, research on how these trends may change as the pandemic progresses could shed light on both the immediate and cumulative benefits of outdoor activities on SWB during times of crisis. Continued exploration of different types of outdoor activities and their health benefits, the “dosage” of nature required to generate benefits [30], as well as motivations for participation in such activities would also be valuable, both during times of stress and times of relative normalcy. As recalled measures of mood, emotions, and SWB can diminish in intensity and become unstable over longer periods of time [80,111], longitudinal studies that feature moment-in-time SWB assessments and integrate other measures of psychological well-being could facilitate tracking of mental health outcomes. Studies focused on other outcomes such as physical health would also contribute to our understanding of the impacts of pandemics and broader global change events. Additionally, the incorporation of qualitative methods in future studies may provide a deeper understanding of the salutogenic aspects of outdoor activity participation not evident in self-report survey responses.

## 5. Conclusions

This study provides evidence that raises concerns regarding declines in adolescent SWB and mental health associated with the COVID-19 pandemic [15,16]. However, results also demonstrate the potential effectiveness of outdoor activity-based interventions in promoting improved SWB for all adolescents regardless of their demographic background [26]. In addition to highlighting the importance of engaging in outdoor activities during COVID-19, this study also illuminates the potential value of outdoor activities as a proactive means of building resilience to stressors associated with future public health challenges and other global change events. As the magnitude and frequency of global crises increases [1], adolescents will face ongoing exposure to stressors that negatively impact their SWB. Facilitating adolescent participation in outdoor activities through policy and infrastructure development, particularly activities that provide opportunities for exposure to nature, physical activity, and social interaction, can be a key step in promoting adolescent health and resiliency during times of crisis.

## Figures and Tables

**Table 1:** Principal component factor analysis for pre COVID-19 pandemic subjective well-being items.

Item	Subjective Well-Being Means	Subjective Well-Being Factor Loadings
Subjective well-being scale	2.21	
Cheerful and in good spirits	2.25	0.87
Calm and relaxed	2.13	0.84
Active and full of energy	2.60	0.82
Interested and curious about the world around me	2.19	0.80
Eigenvalue		2.78
% of variance explained		70%
Chronbach’s alpha		0.85

Response scale items include: At no time = 0, Some of the time = 1, Most of the time = 2, All of the time = 3.

**Table 2:** Principal component factor analysis of adolescent pre COVID-19 pandemic outdoor activity participation items.

Item	Activity Means	Nature-Based Factor	Outdoor Play Factor
Nature-based Activity Scale	0.68		
Paddling (canoeing, kayaking)	0.51	0.76	0.21
Hunting	0.36	0.76	0.03
Camping	0.71	0.73	0.21
Fishing	0.66	0.72	0.16
Wildlife viewing	0.81	0.70	0.24
Hiking	0.82	0.67	0.28
Collecting (flowers, bugs, rocks, feathers, shells, leaves, seeds)	0.71	0.64	0.27
Playing in the woods (building forts, playing games in the woods)	0.88	0.55	0.50 *
Outdoor Play Activity Scale	1.20		
Bicycling outside	1.22	0.23	0.76
Going for walks or runs outside	1.36	0.18	0.74
Playing sports outside	1.42	0.05	0.71
Swimming outside	1.16	0.17	0.67
Skating (skateboard, rollerblades, scooter)	0.84	0.42 *	0.67
Eigenvalues		5.64	1.54
% of variance explained		43%	12%
Chronbach’s alpha		0.88	0.78

Response scale items included: Never = 0, Every now and then = 1, Often = 2. * Cross-loaded items.

**Table 3:** Sample demographics (*N* = 624).

Variable	Categories	*N*	*%*
Gender	Male	306	49.0%
	Female	314	50.3%
	Non-binary	3	0.5%
Race	White	373	59.8%
	Black	71	11.4%
	Hispanic	78	12.5%
	Asian/Pacific Islander	42	6.7%
	Native American	6	1.0%
	Other	6	1.0%
	Two or more races	45	7.2%
Age	10 years	78	12.5%
	11 years	70	11.2%
	12 years	63	10.1%
	13 years	79	12.7%
	14 years	77	12.3%
	15 years	53	8.5%
	16 years	81	13.0%
	17 years	76	12.2%
	18 years	47	7.5%
Community	Rural area	126	20.2%
	Small city or town	126	20.2%
	Suburb near a large city	228	36.5%
	Large city	144	23.1%
Region	South	252	40.4%
	West	136	21.8%
	Midwest	106	17.0%
	Northeast	130	20.8%

The category “prefer not to answer” is not included in this table for gender, race, and income resulting in the % for those categories not adding up to 100.

**Table 4:** Paired sample *t*-tests for pre and post COVID-19 pandemic subjective well-being scores, mental health scores, and outdoor activity scores.

Variable	Pre COVID-19		Post COVID-19		Paired *t* Test	
	Mean	SD	Mean	SD	*t*	*p*
Subjective well-being	2.21	0.616	1.75	0.750	14.870	<0.001
Mental health	4.31	0.798	3.92	0.965	10.919	<0.001
Outdoor play activities	1.20	0.545	0.68	0.566	18.333	<0.001
Nature-based activities	0.68	0.540	0.41	0.492	13.526	<0.001
Outdoor family activities	1.38	0.636	1.02	0.799	10.156	<0.001
General Outdoor Activities	3.68	1.174	2.89	1.453	11.819	<0.001

Response scale items for SWB included: At no time = 0, Some of the time = 1, Most of the time = 2, All of the time = 3. Response scale items for Mental health included: Terrible = 1, Poor = 2, Average = 3, Good = 4, Excellent = 5. Response scale items for outdoor activity groups included: Never = 0, Every now and then = 1, Often = 2. Response scale items for general outdoor activities included: Less than one time per month = 0.25, 1–2 times per month = 0.5, 1 time per week = 1, 2–4 times per week = 3, 5 or more times per week = 5. All *t*-tests were significant after Bonferroni correction to family-wise error rates (*p* = 0.008) [86].

**Table 5:** Linear Regression depicting factors associated with change in subjective well-being scores pre and post COVID-19 for adolescents in the Unites States (*n* = 624).

Difference in SWB Score (Post-Pre)	*B*	Standard Error	Standard Beta	*p*
Pre COVID-19 SWB score	−0.44	0.04	−0.35	0.000 ***
Pre COVID-19 participation in play-based activities	0.30	0.08	0.21	0.000 ***
Pre COVID-19 participation in nature-based activities	−0.08	0.07	−0.06	0.271
Pre COVID-19 participation in outdoor family activities	−0.02	0.05	−0.01	0.714
Change in play-based activity participation during COVID-19	0.44	0.07	0.40	0.000 ***
Change in nature-based activity participation during COVID-19	0.21	0.09	0.14	0.016 *
Change in outdoor family activity participation during COVID-19	0.06	0.04	0.07	0.128
Gender	−0.01	0.05	−0.01	0.804
Age	−0.00	0.01	−0.00	0.941
Income	0.00	0.02	0.00	0.913
Race: White (reference group)				
Black	0.03	0.08	0.01	0.713
Hispanic	0.06	0.08	0.02	0.478
Asian/Pacific Islander	0.02	0.10	0.01	0.832
Native American	−0.31	0.25	−0.04	0.219
Other	−0.29	0.25	−0.04	0.246
Prefer not to answer	−0.20	0.35	−0.02	0.572
Identify as more than one race	0.02	0.10	0.01	0.851
Community type: Suburbs near a large city (reference group)				
Rural area	−0.07	0.07	−0.04	0.313
Small city or town	0.00	0.07	0.00	0.974
Large city	−0.10	0.07	−0.05	0.141
Geographic region: South (reference group)				
West	−0.05	0.07	−0.03	0.414
Midwest	−0.03	0.07	−0.01	0.723
Northeast	0.03	0.07	−0.01	0.687
Intercept	0.59	0.14		0.000 ***

All change scores represent average post-pre scores. Reference groups were selected based on the largest categories for each respective variable. Gender: Male = 0, Female = 1, Non-binary = 2, Prefer not to answer = 4. Age: 10 = 1, 11 = 2, 12 = 3, 13 = 4, 14 = 5, 15 = 6, 16 = 7, 18 = 9. Income: Less than USD 30,000 = 1, USD 30,000–USD 49,999 = 2, USD 50,000–USD 74,999 = 3, USD 75,000–USD 99,999 = 4, USD 100,000–USD 149,999 = 5, USD 150,000 or more = 6, Prefer not to answer = 7. *N* = 624, *R*^2^ = 0.421, Adjusted R^2^ = 0.399, * *p* ≤ 0.05; *** *p* ≤ 0.001.

## Data Availability

The data presented in this study are openly available from the Dryad Data Repository at DOI: 10.5061/dryad.d2547d821.
